# The Pore-Forming Toxin β hemolysin/cytolysin Triggers p38 MAPK-Dependent IL-10 Production in Macrophages and Inhibits Innate Immunity

**DOI:** 10.1371/journal.ppat.1002812

**Published:** 2012-07-19

**Authors:** Magali Bebien, Mary E. Hensler, Suzel Davanture, Li-Chung Hsu, Michael Karin, Jin Mo Park, Lena Alexopoulou, George Y. Liu, Victor Nizet, Toby Lawrence

**Affiliations:** 1 Centre d'Immunologie de Marseille-Luminy (CIML), Aix-Marseille University, UM2, Marseille, France; 2 Institut National de la Santé et de la Recherche Médicale (INSERM), U1104, Marseille, France; 3 Centre National de la Recherche Scientifique (CNRS), UMR7280, Marseille, France; 4 Department of Pediatrics and Skaggs School of Pharmacy and Pharmaceutical Sciences, University of California San Diego, School of Medicine, La Jolla, California, United States of America; 5 Department of Pharmacology, University of California San Diego, School of Medicine, La Jolla, California, United States of America; 6 Cutaneous Biology Research Center, Massachusetts General Hospital, Charlestown, Massachusetts, United States of America; 7 Immunobiology Institute, Cedars-Sinai Medical Center, University of California Los Angeles, School of Medicine, Los Angeles, California, United States of America; University of Birmingham, United Kingdom

## Abstract

*Group B Streptococcus* (GBS) is a leading cause of invasive bacterial infections in human newborns and immune-compromised adults. The pore-forming toxin (PFT) β hemolysin/cytolysin (βh/c) is a major virulence factor for GBS, which is generally attributed to its cytolytic functions. Here we show βh/c has immunomodulatory properties on macrophages at sub-lytic concentrations. βh/c-mediated activation of p38 MAPK drives expression of the anti-inflammatory and immunosuppressive cytokine IL-10, and inhibits both IL-12 and NOS2 expression in GBS-infected macrophages, which are critical factors in host defense. Isogenic mutant bacteria lacking βh/c fail to activate p38-mediated IL-10 production in macrophages and promote increased IL-12 and NOS2 expression. Furthermore, targeted deletion of p38 in macrophages increases resistance to invasive GBS infection in mice, associated with impaired IL-10 induction and increased IL-12 production *in vivo*. These data suggest p38 MAPK activation by βh/c contributes to evasion of host defense through induction of IL-10 expression and inhibition of macrophage activation, a new mechanism of action for a PFT and a novel anti-inflammatory role for p38 in the pathogenesis of invasive bacterial infection. Our studies suggest p38 MAPK may represent a new therapeutic target to blunt virulence and improve clinical outcome of invasive GBS infection.

## Author Summary

Our studies show β hemolysin/cytolysin (βh/c) from *Group B Streptococcus* (GBS), inhibits the activation of macrophages and the innate immune response to GBS. We show that βh/c triggers activation of mitogen activated protein kinase (MAPK) in GBS-infected macrophages leading to expression of the anti-inflammatory cytokine interleukin (IL)-10 and the suppression of genes required for effective anti-bacterial immunity. Furthermore, mice deficient in MAPK activation, specifically in macrophages, show increased resistance to invasive GBS infection. Our data describe a new role for a PFT in the evasion of host immunity that may have significant impact on the pathogenesis of invasive bacterial infections, and suggest targeting the signaling pathways triggered by PFTs in immune cells could increase innate immunity and host resistance.

## Introduction

The pore-forming toxin (PFT) β hemolysin/cytolysin (βh/c) is a major virulence factor for *Group B streptococcus* (GBS), which is generally attributed to its cytolytic functions that aid invasion and avoid phagocytic killing [1]. However, recent data have shown PFTs can act as pathogen associated molecular patterns (PAMP), triggering TLR or NLR pathways in phagocytes [2], [3], [4], suggesting PFTs may have immunomodulatory properties. Despite the central role for βh/c in bacterial pathogenesis, little is known about how mammalian cells respond to this toxin and its effects on host immune signaling. The molecular basis of the host response to βh/c, is critical to understanding the pathogenesis of invasive infections caused by GBS, and may reveal new therapeutic targets to blunt virulence and improve clinical outcome.

Here we used isogenic mutant bacteria to evaluate the role of βh/c in the host response to GBS and particularly on macrophage activation. Macrophages are critical effectors of host defense against bacterial infection [5]; as professional phagocytes, they eliminate bacteria through the coordinate activation of phagocyte oxidase (Nox2), inducible nitric oxide synthase (NOS2), antimicrobial peptides and proteases. Macrophages are also major sources of pro-inflammatory and immunoregulatory cytokines in response to infection, including tumor necrosis factor (TNF)-α, interleukin (IL)-1 and IL-12, and have an important role in orchestrating the innate and adaptive immune response. However, macrophages are also important effectors for the resolution of inflammation, through the production of anti-inflammatory cytokines such as IL-10 and transforming growth factor (TGF)-β, which is critical to limit damage to host tissues [6]. The molecular pathways that regulate the anti-inflammatory functions of macrophages are not clearly understood. To evade host defense, pathogens may hijack anti-inflammatory signaling pathways in macrophages that dampen the immune response and resolve inflammation.

Macrophage activation by bacteria occurs through pattern recognition receptors (PRR) that include membrane associated Toll-like receptors (TLR) and cytoplasmic NOD-LRR containing proteins (NOD-like receptors; NLR) [7]. These receptors recognize specific molecular patterns in bacteria and are coupled to signaling pathways that trigger the release of pro-inflammatory cytokines to amplify inflammation and stimulate adaptive immune responses. The major signaling pathways triggered by TLR and NLR activation by bacteria are the nuclear factor (NF)-κB pathway, interferon regulatory factors (IRFs), and the mitogen-activated protein (MAP) kinases, particularly Jun N-terminal Kinase (JNK), p38 and extracellular-regulated kinase (ERK) [7]. These pathways control the inducible transcription of pro-inflammatory and immunoregulatory genes that dictate the host response. Many bacterial virulence factors have been shown to modulate these signaling pathways in macrophages to evade innate and adaptive immunity through interference with cytokine production and/or induction of apoptosis [8].

Recent work has shown the potential for bacterial PFTs, such as *Streptococcus pneumoniae* pneumolysin, *Bacillus anthracis* anthrolysin O (ALO), and *Staphylococcus aureus* α-toxin to activate macrophages through TLRs [2], [9] or NLRs [4] and affect downstream signaling pathways. However, the consequences of PFT-mediated effects on macrophage activation in disease pathogenesis are poorly understood, and it is not yet clear whether on balance they serve to benefit the pathogen or the host. In the present study, we couple bacterial and mammalian genetic tools to examine the influence of the PFT β-hemolysin/cytolysin (βh/c) produced by GBS on macrophage activation and the host response *in vitro* and *in vivo*. Our results show βh/c-mediated MAPK activation is linked to innate immune evasion and represents a novel virulence mechanism attributed to bacterial PFT.

## Results

### β hemolysin/cytolysin inhibits macrophage killing activity and induction of IL-12 and NOS2 expression at sub-lytic concentrations

**Figure 1:**
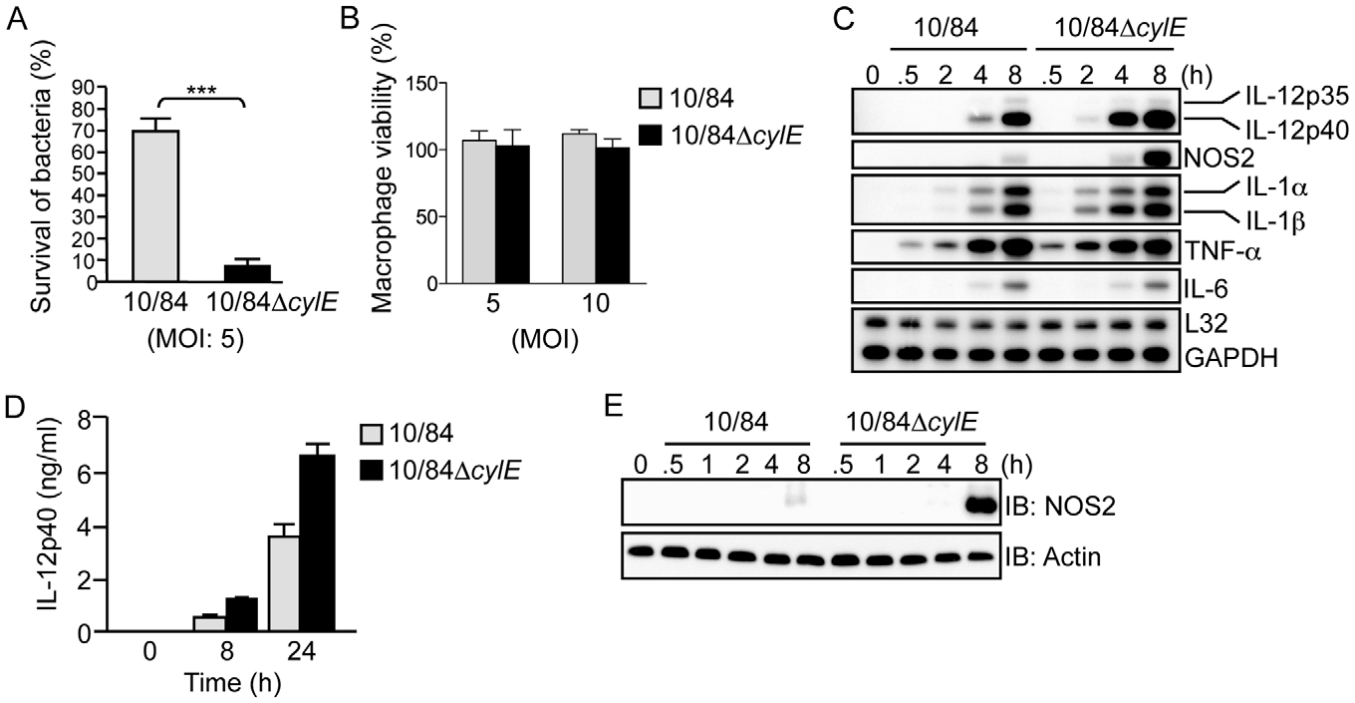
β hemolysin/cytolysin inhibits macrophage killing activity and induction of IL-12 and NOS2 expression at sub-lytic concentrations. **A**. Survival of wt (10/84) and βh/c mutant (10/84Δ*cylE*) GBS bacteria (multiplicity of infection; MOI) in primary macrophages was determined using an *in vitro* killing assay, % survival is plotted as mean ± sem of *n* = 5. **B**. Macrophage viability was assessed by MTT colorimetric assay after infection with 10/84 and 10/84Δ*cylE* bacteria at the indicated MOI, % viability is plotted as mean ± sem of 4 replicates, representative data is shown from at least 2 independent experiments. **C**. Total RNA was isolated from macrophages infected with 10/84 and 10/84Δ*cylE* bacteria (MOI: 5) at the indicated time points, and analyzed by ribonuclease protection assay (RPA) using a multi-probe template set to measure cytokine mRNA expression. Representative data from at least 3 independent experiments is shown. **D**. IL-12p40 production was measured in cell-culture supernatants from macrophages infected with 10/84 and 10/84Δ*cylE* bacteria for the indicated time points by ELISA. Data is represented as mean ± sem of 4 replicates, representative data from at least 3 independent experiments is shown. **E**. Protein extracts were prepared from macrophages infected with 10/84 and 10/84Δ*cylE* bacteria at the indicated time points and NOS2 expression measured by immunoblotting (IB), actin was used as a loading control. Representative data from at least 3 independent experiments is shown.

*β hemolysin/cytolysin* (βh/c) is an important virulence factor for GBS, as demonstrated in numerous animal models of systemic infection [10]. We have used the invasive wild-type (wt) GBS clinical isolate (serotype V strain NCTC 10/84) and its isogenic βh/c-deficient mutant 10/84Δ*cylE* [11], to study the role of βh/c in macrophage activation. Infection of primary mouse macrophages with wt GBS (10/84) and the βh/c mutant (10/84Δ*cylE*), showed dramatically reduced survival of the Δ*cylE* mutant 60 min after infection compared to the wt strain (Fig. 1A), however this was not associated with any effects on macrophage viability (Fig. 1B), ruling out the cytolytic action of βh/c as a mechanism for inhibition of macrophage activity. Previous studies have also shown no difference in uptake of Δ*cylE* mutant bacteria by macrophages or neutrophils [11], suggesting that βh/c inhibits the intracellular killing of GBS in macrophages. In parallel experiments, we isolated RNA from GBS-infected macrophages and measured cytokine gene expression by ribonuclease protection assay (RPA). Despite the presence of significantly lower numbers of bacteria in 10/84Δ*cylE*-infected macrophages (almost 8 times less, Fig. 1A), the βh/c-deficient bacteria increased specifically expression of IL-12p40 and NOS2 mRNA, whereas levels of mRNA for other inflammatory cytokines including IL-6 and TNF-α were relatively unchanged (Fig. 1C). Increased production of IL-12p40 and NOS2 protein by 10/84Δ*cylE*-infected macrophages was confirmed by ELISA and immunoblotting, respectively (Fig. 1D,E). Macrophage IL-12 and NOS2 expression are critical factors in antibacterial host defense [5], [12], in particular NOS2 induction by GBS is an important factor for macrophage-mediated killing [13], thus the inhibition of their production by sub-cytolytic concentrations of βh/c may facilitate evasion of the host response and potentially be an important factor in resistance to GBS infection.

### βh/c increases JNK and p38 MAPK activation in GBS-infected macrophages

**Figure 2:**
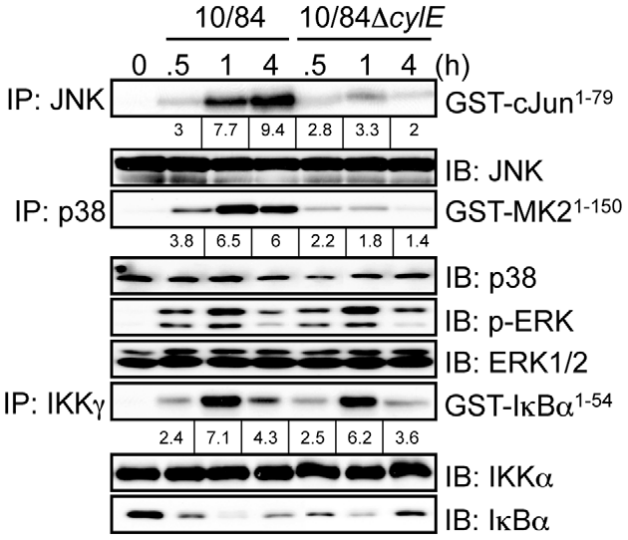
βh/c increases JNK and p38 MAPK activation in GBS infected macrophages. Protein extracts were prepared from macrophages infected with 10/84 and 10/84Δ*cylE* bacteria at the indicated time points. JNK, p38 and IKKγ were immunoprecipitated (IP) and kinase activity was measured by incubation with recombinant substrates; GST-cJun^1–79^, GST-MAPKAPK2 (GST-MK2^1–150^) and GST-IκBα^1–54^, in the presence of ^32^P-γ-ATP. Substrates were resolved by SDS–PAGE and phosphorylation quantified by radioisotope imaging using a phosphor imager and autoradiography, fold change over control is indicated underneath the respective bands. Equal loading of kinase IP was confirmed by IB for the respective kinases. Protein extracts were also analyzed by IB for phospho-ERK, ERK and IκBα expression. Representative data from at least 3 independent experiments is shown.

IL-12 and NOS2 expression in macrophages is regulated by NF-κB and MAPK activation [14], [15]. To study the effect of βh/c on these signaling pathways, we infected macrophages with wt or Δ*cylE* GBS and measured activation of IκB kinase (IKK), the kinase regulating NF-κB activation, and the MAPKs p38 and JNK. Protein extracts were prepared from infected macrophages at the indicated time points; p38, JNK, and IKK were immunoprecipitated (IP) from extracts using specific antibodies, and the immune-complexes incubated with recombinant substrates in the presence of radioactive ATP (^32^P-γ-ATP). Substrates were resolved by SDS-PAGE and phosphorylation measured by autoradiography. In the case of ERK, phospho-specific antibody was used to measure activation of the kinase in protein extracts. Macrophages infected with wt GBS showed robust activation of IKK, JNK, ERK and p38 between 30–60 min after infection (Fig. 2). In comparison, macrophages infected with the Δ*cylE* bacteria showed dramatically reduced JNK and p38 activation, while IKK and ERK activation were similar to wt (Fig. 2). These data suggested βh/c specifically increases JNK and p38 activity in GBS-infected macrophages.

As shown in Fig. 1A, Δ*cylE* bacteria are much more readily killed by macrophages, but despite almost 8 times less intracellular bacteria, expression of IL-12 and NOS2 was increased in 10/84Δ*cylE*-infected macrophages (Fig. 1C). However, this did not correlate with increase NF-κB or MAPK activation, but in fact significantly reduced JNK and p38 activity (Fig. 2), suggesting that p38 and JNK activation by βh/c may be associated with inhibition, rather than activation, of IL-12 and NOS2 expression.

**Figure 3:**
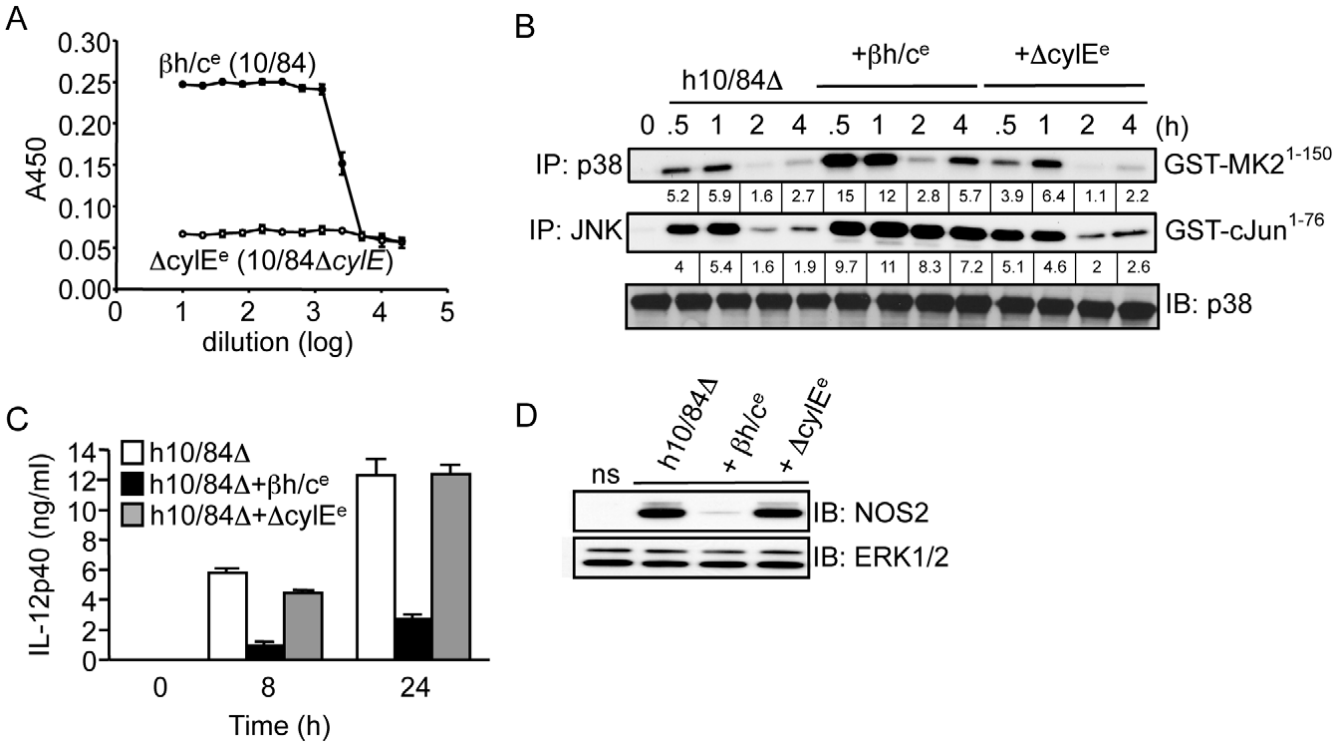
Partially purified βh/c triggers MAPK activation and inhibits IL-12 and NOS2 expression in macrophages. **A**. βh/c extract was prepared from wt GBS (10/84; βh/c^e^) and a mock extract from 10/84Δ*cylE* bacteria (ΔcylE^e^), hemolytic activity measured by sheep red blood cell (RBC) lysis assay. **B**. Macrophages were stimulated with heat-killed 10/84Δ*cylE* bacteria (h10/84Δ, MOI: 20) in the presence of βh/c^e^ or ΔcylE^e^ at a dilution of 1∶200, and protein extracts prepared at the indicated time points. JNK and p38 activity were measured by IP kinase assay, as described in Fig. 2. IB for p38 was used as loading control. Representative data from at least 2 independent experiments is shown. **C**. IL-12p40 was measured in cell culture supernatants by ELISA at 8 and 24 h after stimulation of macrophages with h10/84Δ in the presence of βh/c^e^ or ΔcylE^e^. Data is represented as mean ± sem of 4 replicates, representative data from at least 3 independent experiments is shown. **D**. Protein extracts were prepared from macrophages 8 h after stimulation with h10/84Δ in the presence of βh/c^e^ or ΔcylE^e^ and NOS2 expression measured by IB, ERK expression was used as a loading control. Representative data from at least 3 independent experiments is shown.

We next attempted to reconstitute the activity of βh/c using a purified form of the toxin. Unfortunately, βh/c has not yet been successfully purified to homogeneity and therefore it is not possible to use recombinant purified βh/c. However, we have generated a cell-free extract from wt GBS that reconstitutes βh/c activity (βh/c^e^) and a control extract from Δ*cylE* bacteria (ΔcylE^e^), which has no hemolytic activity (Fig. 3A). We confirmed that βh/c^e^, and not the mock extract prepared from Δ*cylE* bacteria, specifically activates JNK and p38 in macrophages (Fig.S1A). The absence of IKK activation by either extract confirmed no contamination with TLR ligands. Furthermore, in macrophages infected with Δ*cylE* bacteria βh/c^e^ was able to reconstitute p38 and JNK activity similar to wt GBS, without effect on IKK (Fig.S1B). To control for differences in killing of wt and Δ*cylE* bacteria, we stimulated macrophages with heat-killed Δ*cylE* bacteria (h10/84Δ) in the presence of βh/c^e^ or the mock extract (ΔcylE^e^); addition of βh/c^e^ specifically increased p38 and JNK activity which was associated with inhibition of IL-12 and NOS2 expression, whereas the mock extract had no effect (Fig. 3B,C,D). These experiments corroborate our studies with live GBS and βh/c-deficient bacteria, and confirm the inhibitory effects of βh/c on IL-12 and NOS2 expression in macrophages is not due to the differential killing of wt and Δ*cylE* bacteria. The effects of βh/c^e^ on IL-12 and NOS2 expression were again evident at sub-lytic concentrations (Fig.S1C,D), as observed with live GBS infection (Fig. 1B). These studies revealed a novel and surprising association between MAPK activation by βh/c and inhibition of IL-12 and NOS2 in GBS-infected macrophages.

### MAPK activation by βh/c is TLR, NOD2 and inflammasome-independent

**Figure 4:**
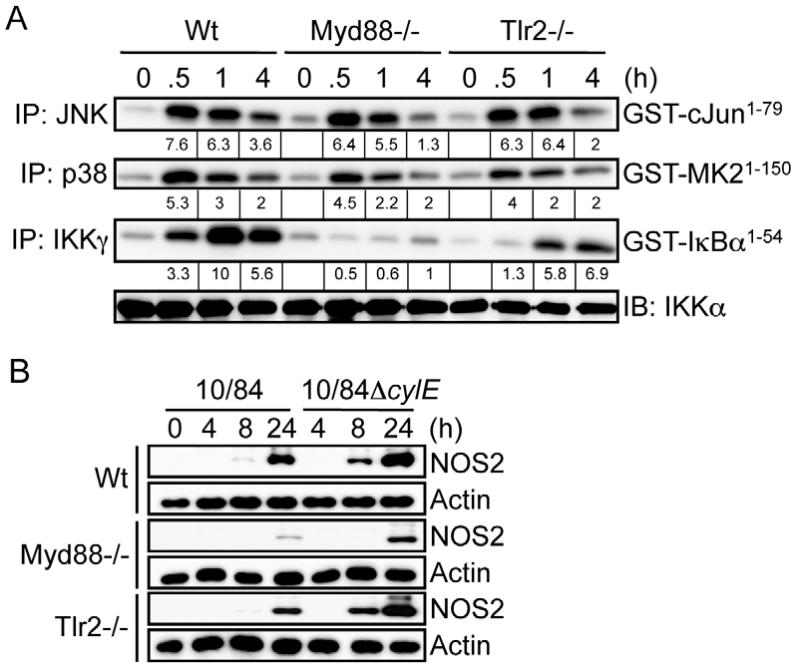
βh/c-mediated JNK and p38 activation is TLR2/MyD88-independent. **A**. Primary macrophages were prepared from wild type (Wt), MyD88 (Myd88) and TLR2 (Tlr2) knockout mice. Macrophages were infected with 10/84 (MOI: 5) and protein extracts prepared at the indicated time points. JNK, p38 and IKK activity were measured by IP kinase assay, as described in Fig. 2. IB for IKKα was used as loading control. **B**. Protein extracts were prepared from Wt, Myd88−/− and Tlr2−/− macrophages infected with 10/84 and 10/84Δ*cylE* bacteria (MOI: 5) at the indicated time points and NOS2 expression measured by IB, actin was used as a loading control. Representative data from at least 2 independent experiments is shown.

Activation of IKK and MAPK by extracellular bacteria occurs through pattern recognition receptors (PRR) that include membrane-associated TLRs and cytoplasmic NLR proteins [7]. Previous studies have suggested certain PFTs may act as TLR ligands [2], to test this hypothesis with βh/c we measured IKK and MAPK activation in GBS-infected macrophages derived from TLR2, TLR4 and MyD88 knockout mice. While the principle ligand for TLR4 is lipopolysaccharide (LPS) from Gram-negative bacteria [7], this PRR has also been reported to be activated by PFTs from Gram-positive bacteria [2]. However, we found no role for TLR4 in either IKK or MAPK activation by GBS, suggesting these observations do not apply to βh/c (Fig.S3A). As a Gram-positive bacteria, GBS is expected to trigger activation of TLR2/MyD88 through peptidoglycan (PGN) as well as TLR9/MyD88 through bacterial DNA [7]. GBS-induced IKK activation was blocked in the absence of MyD88 and partially inhibited in the absence of TLR2 (Fig. 4A), the remaining IKK activity in *Tlr2*−/− cells probably occurs due to TLR9 activation. However, somewhat surprisingly, TLR2 and MyD88 were not required for GBS-induced JNK or p38 activity (Fig. 4A). In addition, we compared infection with wt and Δ*cylE* GBS in *Myd88−/−* and *Tlr2−/−* macrophages. As expected, induction of NOS2 expression was MyD88-dependent (Fig. 2B), however increased NOS2 expression in 10/84Δ*cylE*-infected macrophages was still evident in the absence of MyD88 and TLR2 (Fig. 2B), suggesting inhibition of NOS2 expression by βh/c was mediated independently of TLR2/MyD88 activation.

The NLR protein NOD2 has also been suggested to trigger MAPK activation through recognition of PGN from Gram-positive bacteria [12], and was recently suggested to be important in the host response to α-toxin from *S. aureus* [4], however NOD2 was dispensable for IKK and MAPK activation in GBS-infected macrophages (Fig.S2B). ASC (apoptosis-associated speck-like protein containing a CARD) is a critical component of the caspase-1 inflammasome, that has been reported to be triggered by PFTs from several bacteria [16], [17], [18], however both *Asc1*−/− and caspase-1 deficient (*Casp1*−/−) macrophages showed no defect in GBS-mediated IKK or MAPK activation (Fig.S2C,D), indicating the inflammasome was also not required for βh/c-associated MAPK activation. Furthermore, as shown for TLR2/MyD88 (Fig. 2B), inhibition of NOS2 expression by wild type GBS was independent of NOD2, caspase-1 and ASC (data not shown).

In sum, these data suggest βh/c-associated p38 and JNK activation is independent of known PRR pathways for Gram-positive bacteria including; TLRs, NOD2 and the ASC/Caspase-1 inflammasome. Furthermore, inhibition of NOS2 expression by wild type GBS occurs independently of TLR/NLR signaling, which suggests MAPK activation by βh/c may contribute to virulence rather than pathogen recognition, although we cannot rule out a role for as yet unknown PRR pathways for Gram-positive bacteria in MAPK activation by βh/c.

### βh/c induces p38-dependent IL-10 expression in GBS-infected macrophages

**Figure 5:**
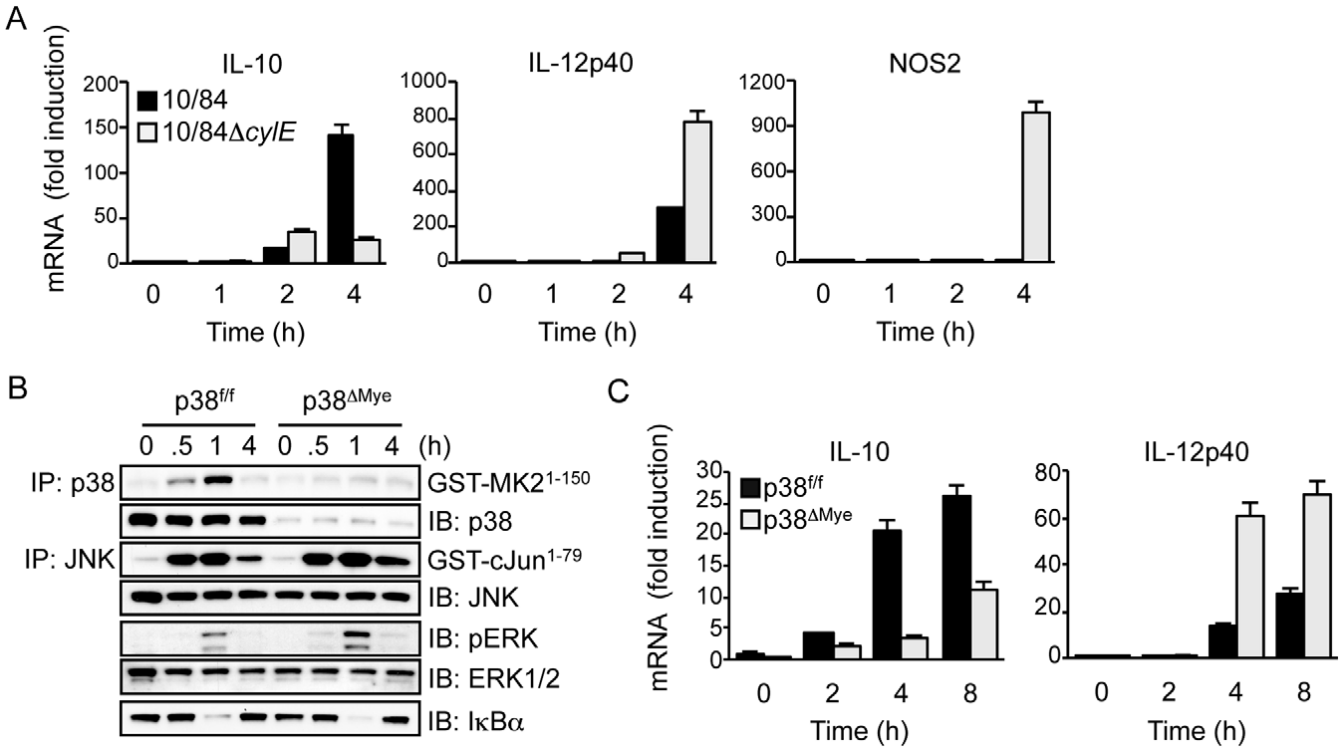
βh/c drives p38-dependent IL-10 expression in GBS-infected macrophages. **A**. Primary macrophages were infected with 10/84 and 10/84Δ*cylE* (MOI 5) and total RNA prepared at the indicated time points. IL-10, IL-12p40 and NOS2 mRNA expression was measured by real-time quantitative PCR (qPCR). Data is expressed as fold induction normalized to cyclophillin mRNA expression and the mean ± sem of 3 replicates is plotted. Representative data is shown from at least 2 independent experiments. **B**. Primary macrophages from p38^f/f^ and p38^ΔMye^ mice were infected with 10/84 (MOI: 5) and protein extracts prepared at the indicated time points. JNK, p38 and IKK activity was measured by IP kinase assay, phospho-ERK (pERK) and IκBα were measured by IB, as described in Fig. 2. **C**. Total RNA was isolated from p38^f/f^ and p38^ΔMye^ macrophages infected with 10/84 at the indicated time points and IL-10 and IL-12p40 mRNA expression was measured by qPCR. Data is expressed as fold induction normalized to cyclophillin mRNA and the mean ± sem of 3 replicates is plotted. Representative data is shown from at least 2 independent experiments.

IL-10 is an important anti-inflammatory cytokine produced by macrophages during infection that strongly inhibits IL-12 and NOS2 expression [19]. It has recently been shown that TLR4-mediated IL-10 expression in macrophages is p38-dependent [20], therefore we tested the role of βh/c-mediated p38 activation in IL-10 expression in GBS-infected macrophages. First we established that GBS-induced IL-10 expression was βh/c-dependent, comparing IL-10 mRNA induction in macrophages infected with wt or Δ*cylE* bacteria, IL-10 expression was strongly induced by wt GBS but not the 10/84Δ*cylE* mutant (Fig. 5A). Furthermore, decreased IL-10 expression upon infection with 10/84Δ*cylE* bacteria correlated with increased IL-12p40 and NOS2 mRNA (Fig. 5A). To confirm IL-10 induction in GBS-infected macrophages was linked to inhibition of IL-12 expression, we infected macrophages from IL-10 knockout mice (*Il10−/−*) with wt GBS; *Il10*−/− macrophages showed a profound increase in IL-12p40 expression after infection with wt GBS, almost 200 fold within 4 h (Fig.S3A); furthermore, addition of an IL-10 receptor-blocking antibody reversed the inhibition of IL-12 expression by βh/c^e^ in macrophages stimulated with heat-killed 10/84Δ*cylE* bacteria (Fig.S3B). These data demonstrate the induction of IL-10 expression in macrophages by βh/c is associated with autocrine inhibition of IL-12 production.

To confirm the role of p38 in GBS-induced IL-10 expression, and repression of IL-12, we used macrophages from myeloid-specific p38 knockout mice (p38^ΔMye^) [20]. These mice have a targeted deletion in p38 restricted to neutrophils and macrophages generated by Cre/*lox*-mediated gene targeting. Biochemical analysis of MAPK and IKK activation in GBS-infected macrophages from p38^ΔMye^ mice confirmed specific ablation of p38 activity with normal activation of JNK, ERK and IKK (Fig. 5B). This was associated with inhibition of IL-10 mRNA induction and a reciprocal increase in IL-12p40 expression (Fig. 5C). The specific increase in IL-12p40 mRNA expression in p38^ΔMye^ macrophages was confirmed by RPA analysis, that showed little change in other pro-inflammatory genes including IL-1β and IL-6 (Fig.S3C). These data demonstrate that GBS-induced IL-10 expression, and inhibition of IL-12, in macrophages is p38-dependent. This suggests βh/c-mediated p38 activation in the context of GBS infection drives IL-10 expression in macrophages and is likely to promote anti-inflammatory effects and evasion of the host response.

### Targeted deletion of p38 in macrophages increases resistance to invasive GBS infection *in vivo*

**Figure 6:**
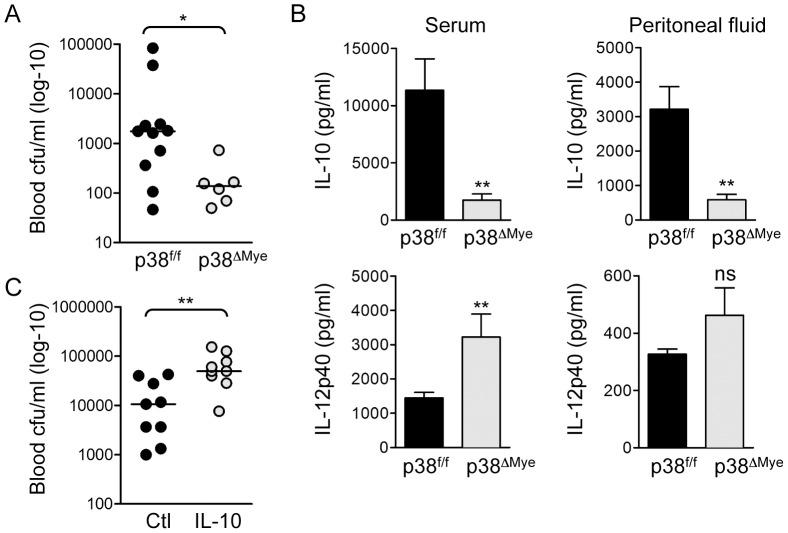
Targeted deletion of p38 in macrophages increases resistance to invasive GBS infection *in vivo*. **A**. p38^f/f^ and p38^ΔMye^ mice were infected i.p. with 5×10^7^ cfu GBS (10/84) and blood cfu was determined after 8 h. The cfu for individual mice is represented in the scatter plot and median indicated by the bar. Statistical analysis was performed using non-parametric Mann Whitney t-test, *p<0.05. **B**. IL-10 and IL-12p40 were measured in serum and peritoneal fluid of GBS infected mice by ELISA. Data is represented as mean ± sem, statistical analysis was performed using Mann Whitney t-test, **p<0.005. **C**. Wt mice were injected i.p. with 400 ng recombinant mouse IL-10 before i.p. infection with 5×10^7^ cfu GBS. Blood cfu was determined 8 h after infection. The cfu for individual mice is represented in the scatter plot and median indicated by the bar. Statistical analysis was performed using non-parametric Mann Whitney t-test, **p<0.005.

To determine the role of p38 in the pathogenesis of invasive GBS infection *in vivo*, we used a mouse model of GBS-induced sepsis [11], [21]. To test the specific role of p38 activation in macrophages after GBS infection we again used p38^ΔMye^ mice [20]. We infected p38^ΔMye^ and littermate control mice (p38^f/f^) with 5×10^7^ cfu GBS (10/84) by intra-peritoneal (i.p.) inoculation, and monitored clearance of bacteria from the blood; p38^ΔMye^ mice showed a significant reduction in blood cfu after 8 h compared with control mice (Fig. 6A), demonstrating that p38 activation in macrophages inhibits killing of GBS *in vivo*. Increased clearance of GBS infection by p38^ΔMye^ mice correlated with significantly reduced levels of IL-10 in serum and peritoneal fluid and reciprocally increased levels of IL-12 (Fig. 6B), suggesting an important role for p38 activity in macrophages for IL-10 production during GBS infection. Reduced expression of IL-10 mRNA was confirmed in peritoneal macrophages isolated from GBS-infected p38^ΔMye^ mice compared with littermate controls (Fig.S4). Furthermore, administration of recombinant IL-10 to GBS-infected mice significantly increased survival of bacteria in the blood (Fig. 6C), demonstrating that IL-10 inhibits killing of GBS *in vivo*. These data suggest p38-medaited IL-10 production by macrophages during GBS infection is a significant factor in host resistance.

### Triggering of p38-mediated IL-10 production in macrophages is not a general property of pore-forming toxins

**Figure 7:**
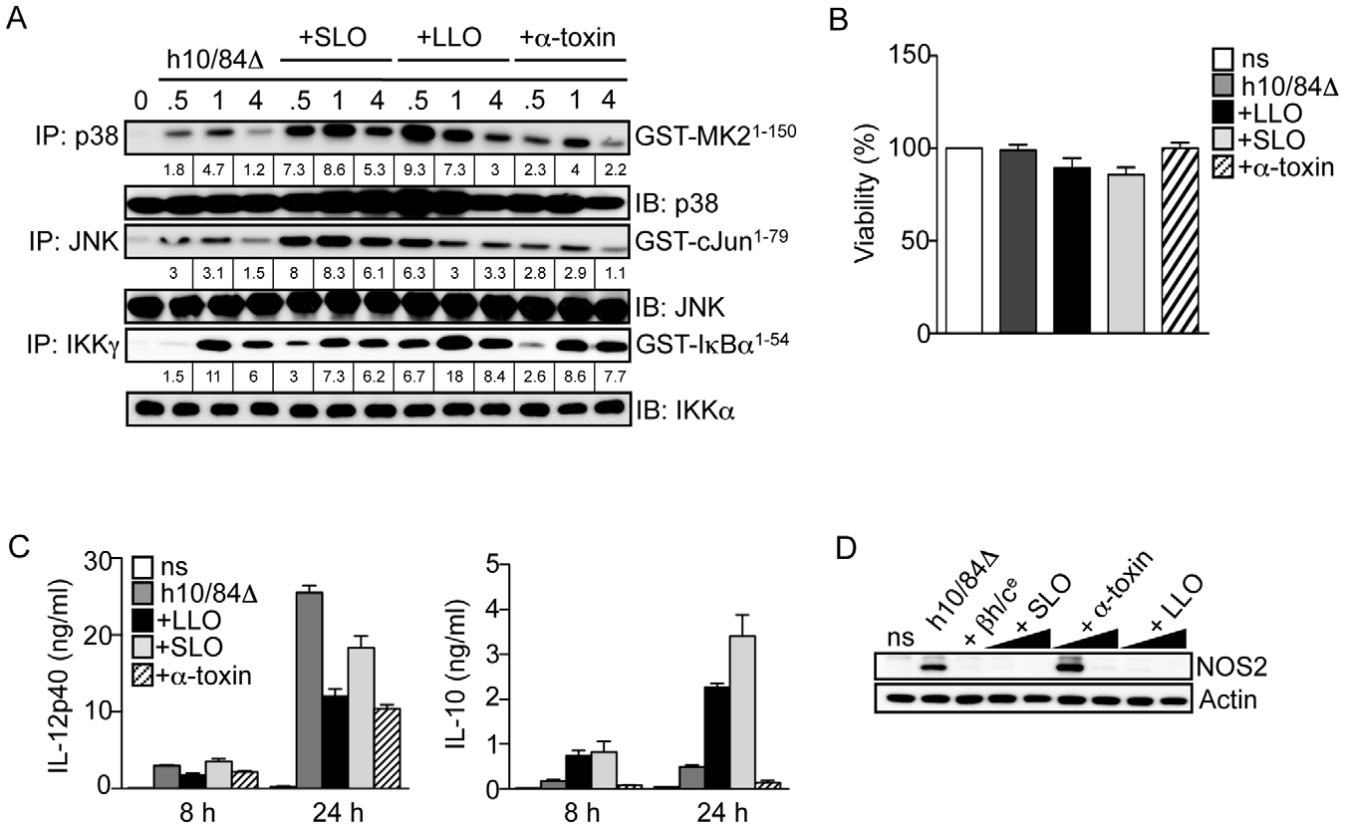
Triggering of p38-mediated IL-10 production in macrophages is not a general property of pore-forming toxins. **A**. Macrophages were stimulated with heat-killed 10/84Δ*cylE* bacteria (h10/84Δ) alone and in the presence of 1.0 µg/ml purified LLO from *L. monocytogenes*, SLO from *S. pyogenes*, or α-toxin from *S. aureus*, and protein extracts prepared at the indicated time points. JNK, p38 and IKK activity was measured by IP kinase assay, as described in Fig. 2. Representative data from at least 2 independent experiments is shown. **B**. Viability of macrophages stimulated with h10/84Δ in the presence of 1.0 µg/ml LLO, SLO or α-toxin for 8 h was measured by MTT assay (ns: non stimulated control), data is represented as mean ± sem of 4 replicates and representative data from at least 2 independent experiments is shown. **C**. IL-12p40 and IL-10 production was measured in cell culture supernatants by ELISA at 8 and 24 h after stimulation with h10/84Δ in the presence of LLO, SLO or α-toxin. Data is represented as mean ± sem of 4 replicates and representative data from at least 2 independent experiments is shown. **D**. Protein extracts were prepared from macrophages 8 h after stimulation with h10/84Δ in the presence of βh/c^e^ (1∶200), LLO (0.1, 1 µg/ml), SLO (0.1, 1 µg/ml) or α-toxin (1, 2 µg/ml), and NOS2 expression measured by IB, actin was used as a loading control.

Finally, we asked if the effects of βh/c on p38-mediated IL-10 expression and repression of NOS2 and IL-12 could be extended to other PFTs from distantly related pathogenic bacteria. We used recombinant listeriolysin O (LLO) from *Listeria monocytogenes*, streptolysin O (SLO) from *Streptococcus pyogenes*, and α-toxin from *S. aureus*. LLO and SLO belong to the family of cholesterol-dependent PFTs that form large pores, α-toxin on the other hand is a β barrel toxin that forms relatively small membrane pores. Only LLO and SLO increased MAPK activation in combination with heat-killed Δ*cylE* bacteria at sub-lytic concentrations (Fig. 7A,B). Although all 3 toxins inhibited IL-12 and NOS2 expression, only in the case of SLO and LLO was this associated with induction of IL-10 (Fig. 7C,D). Since not all the PFTs tested led to increased p38 activation and IL-10 expression in macrophages, we tested the effect of membrane permeabilisation on MAPK activation and IL-10 expression in macrophages stimulated with heat-killed Δ*cylE* bacteria, using the detergent saponin. Addition of saponin alone mildly induced p38 activity in macrophages, however saponin had no effect on p38 activation, or expression if IL-10, in macrophages stimulated with heat-killed Δ*cylE* bacteria (Fig.S5), in contrast to the effects of βh/c, SLO and LLO (Fig. 7).

These data suggest the immunomodulatory properties of βh/c may be shared by some, but not all PFTs, and the triggering of p38-mediated IL-10 production by βh/c is independent of osmotic stress induced by membrane permeabilisation.

## Discussion

Here we used isogenic mutant bacteria to evaluate the role of β hemolysin/cytolysin (βh/c) [22] in the host response to GBS. There was a dramatic inhibition of macrophage-killing activity by βh/c, without effect on macrophage viability. Despite enhanced killing of βh/c-deficient bacteria there was increased expression of NOS2 and IL-12 in infected macrophages, but decreased expression of the anti-inflammatory and immunosuppressive cytokine IL-10. These data suggest βh/c modulates cytokine production in macrophages that would favor survival of the bacteria and contribute to virulence.

Cytokine production by macrophages in response to infection is triggered by engagement of membrane bound TLR and cytosolic NLR receptors by various bacterial products. These receptors are coupled to NF-κB and MAPK signaling pathways that regulate gene expression. To test the effects of βh/c on NF-κB and MAPK signaling, we performed biochemical analysis of IKK (the kinase that regulates NF-κB activation), p38 and JNK activity in GBS-infected macrophages. Somewhat surprisingly we found inhibition of IL-12 and NOS2 expression by βh/c was associated with increased p38 and JNK activation, but no effect on IKK/NF-κB activity. Recent studies using purified toxins have suggested PFTs can directly trigger TLR and NLR activation in macrophages [2], [3], [4]. To examine the role of TLR/NLR signaling in βh/c mediated MAPK activation we used macrophages derived from transgenic mice deficient in different TLR/NLR signaling pathways. TLR-mediated IKK/NF-κB and MAPK activation by extracellular bacteria is regulated by the adaptor protein MyD88 [7], NLR-mediated inflammasome activation can also trigger IKK/NF-κB and MAPK through autocrine production of IL-1β, that also signals through MyD88 [23]. We found that βh/c-associated MAPK activation was unaffected in MyD88 deficient macrophages, whereas activation of IKK was MyD88-dependent as expected. We also confirmed that wild type GBS did not activate MAPK through TLR2 or TLR4 using macrophages from respective knockout mice. IKK and MAPK activation by the NLR NOD2 has been reported through activation of the kinase RIP2/RICK/CARDIAK [7], however we found no role for NOD2 in βh/c-associated MAPK activation using macrophages from NOD2 deficient mice. Finally, we tested if the inflammasome was involved, although a role for autocrine IL-1β production was ruled out using MyD88 deficient macrophages. Both ASC and caspase-1, which are essential components of the inflammasome, had no role in βh/c-associated MAPK activation using macrophages derived from respective gene targeted mice. These experiments using isogenic mutant bacteria show βh/c-associated MAPK activation in GBS-infected macrophages occurs independently of TLR/NLR signaling.

GBS βh/c is a surface associated PFT with broad spectrum cytolytic activity [24], however due to inherent instability of soluble βh/c, the toxin has not yet been purified to homogeneity. Cell free extracts of GBS βh/c can be prepared using appropriate high molecular weight carrier molecules, such as albumin or complex carbohydrate [3]. To further investigate the effects of βh/c on macrophage activation we used a stabilized cell free extract of GBS βh/c that maintains hemolytic/cytolytic activity (βh/c^e^) [25]. This extract was able to reconstitute the effects of wt GBS on MAPK activation and cytokine production by macrophages when added with βh/c-deficient bacteria. To test for similar effects with PFTs from distantly related bacteria, we used recombinant toxins from *S. aureus* (α-toxin), *S. pyogenes* (SLO) and *L. monocytogenes* (LLO). SLO and LLO recapitulated the effects of βh/c on p38 activation and regulation of IL-12, NOS2 and IL-10 expression. *S. aureus* α-toxin showed similar ability to inhibit IL-12 expression but did not increase p38 activation or expression of IL-10, α-toxin belongs to a distinct family of β-barrel PFTs that form relatively small pores compared to the cholesterol-dependent PFTs LLO and SLO that form very large pores [26], and βh/c that also forms large pores [3]. Other studies have also shown differences in the cellular response to these different classes of PFT [27], [28] and the differences in their mode of action may give us further insight into the mechanism of MAPK activation by βh/c and other PFTs. This is a topic of current investigation.

A conserved property of PFTs is membrane permeabilisation, and osmotic stress induced by LLO has been suggested as a mechanism for MAPK activation in epithelial cells [29]. In these studies the authors compared the effects of the detergent saponin to recombinant LLO. However, in our studies membrane permeabilisation with saponin only mildly affected p38 activity and was not associated with induction of IL-10 expression in macrophages, suggesting osmotic stress is not the mechanism for p38 activation by βh/c in this context. Therefore, MAPK activation by βh/c appears to be independent of TLR/NLR signaling or osmotic stress, and may involve novel signaling intermediates recruited by the specific action of this toxin or a component of the toxin itself. Further investigations are required to delineate the molecular mechanisms of MAPK activation by βh/c, which will be greatly facilitated by the purification and production of recombinant βh/c.

Many important human pathogens including; pneumococcus, *L. monocytogenes*, *S. pyogenes*, GBS and *S. aureus*, are known to activate MAPK in mammalian cells [30], [31], [32], [33], and in the case of *L. monocytogenes*, this has been linked to the specific action of the PFT LLO [34]. But the role of MAPK in pathogenesis of these invasive bacteria has not been addressed. Here we show that βh/c induces expression of the anti-inflammatory cytokine IL-10 in GBS-infected macrophages through activation of p38. Furthermore, using macrophage-specific p38 knockout mice we show that p38-dependent IL-10 production by macrophages impairs host defense during GBS infection *in vivo*. Recent studies have shown IL-10 expression by macrophages in response to bacterial lipopolysaccharide (LPS) is p38-dependent [20], [35], but this is the first description of p38-dependent IL-10 expression in the context of infection and attributed to bacterial virulence. Other studies have shown induction of IL-10 production by virulence factors of bacterial pathogens including Bordetella [36] and *Yersina pestis* [37], that is linked with pathogenesis and evasion of host immunity, and it would be interesting to test the p38 dependence of IL-10 expression in these contexts.

It should be noted that anthrax lethal factor, a virulence factor of *Bacillus anthracis*, induces macrophage apoptosis through inhibition p38-signaling downstream of TLR engagement (32), which in this case is linked to bacterial virulence. *B. anthracis* is an obligate intracellular pathogen, whereas GBS occupies an extracellular niche, thus the role of p38 activation may be quite different in this context. Furthermore, in our experimental conditions βh/c does not induce macrophage apoptosis (data not shown), although at higher concentrations βh/c will readily induce macrophage lysis. Our studies also show clearly that βh/c-mediated MAPK activation is independent of TLR signaling and is likely to involve distinct signaling intermediates. The consequences of TLR and PFT-mediated p38 activation may be quite different, for example; TNF-α expression in response to TLR ligands is p38-dependent [38], however in our studies, PFT-mediated p38 activation is not associated with increased TNF-α production. Interestingly, it has been shown that HIV Tat protein also induces p38-dependent IL-10 expression in infected monocytes independently of TNFα production [39].

In summary, our studies show the PFT βh/c triggers p38-dependent IL-10 expression and suppression of NOS2 and IL-12 in macrophages during GBS infection, which is linked with evasion of innate immunity. This represents a novel virulence mechanism for a PFT and an unexpected anti-inflammatory function for p38 in the pathogenesis of invasive bacterial infections. Macrophage NOS2 and IL-12 expression are critical for host resistance to bacterial infection, and their inhibition by βh/c may significantly impact the pathogenesis of GBS infections. Of note, impaired production of IL-12 by human neonatal macrophages has been specifically linked to increased susceptibility to GBS infection [40]. Since GBS represents a major cause of invasive infections in human newborns, the inhibition of IL-12 expression by βh/c may be particularly important for virulence in this population. Our studies suggest specific targeting of p38 activation in macrophages could increase innate resistance to GBS infection in susceptible populations such as the newborn, pregnant women, and immune-compromised individuals.

## Materials and Methods

### Mice

C57Bl6 mice and gene-targeted mice on the same genetic background were used for all experiments.

### Bone marrow derived macrophages (BMDM)

Bone marrow-derived macrophages were generated as described previously [21]. Briefly, femurs and tibiae from mice aged 8 to 10 weeks were flushed and cells collected by centrifugation at 450×g for 5 min at 4°C. Cells were then re-suspended in DMEM supplemented with L-glutamine (2 mM), penicillin (100 U/ml)/streptomycin (100 µg/ml), 10% heat-inactivated FBS and 10 ng/ml recombinant mouse M-CSF (Peprotech) and cultured at a density of 1×10^6^ cells/ml in non-tissue culture treated plastic dishes (BD Pharmingen) at 37°C and 5% CO_2_. After 7 days, adherent cells were collected and cell viability was measured using a ViCell XR Counter (Beckman Coulter). BMDM were then diluted in complete DMEM containing 10 ng/ml M-CSF and plated in culture dishes for further experiments.

### Bacteria

NCTC 10/84 hemolytic GBS serotype V is a clinical isolate from neonatal sepsis [22] and the corresponding non-hemolytic isogenic βh/c-deficient mutant NCTC:*cylE*Δ*cat* was generated by allelic replacement [22]. Here we use the abbreviated designations 10/84 and 10/84Δ*cylE*, respectively. Bacteria were grown in Todd-Hewitt broth (THB) or on Todd-Hewitt agar (THA) (Difco). Both strains grew equally well under the conditions used in the experiments. Heat-killed 10/84Δ*cylE* bacteria (designated hereafter h10/84Δ) were obtained by heating logarithmic-phase 10/84Δ*cylE* (10^8^ cfu/ml; OD_600 nm_ = 0.4) for 30 min at 80°C. Prior to infection with bacteria at the indicated MOI, BMDM were maintained in antibiotic free-medium plus 10% FBS in the presence of M-CSF. After 30 min of infection, bacteria were removed and fresh culture medium containing penicillin-streptavidin and gentamycin (50 µg/ml) was then added to kill extracellular bacteria. When using heat-killed bacteria, BMDM were maintained in complete DMEM containing M-CSF and antibiotics. For *in vivo* experiments logarithmic-phase bacteria were prepared and washed 3 times with sterile PBS. Bacteria were diluted in PBS and injected i.p. at the indicated dose.

### Preparation of GBS hemolysin extracts

Partially purified hemolysin extracts from 10/84 and 10/84Δ*cylE* were prepared as previously described [25]. Briefly, 10/84 and 10/84Δ*cylE* were grown in THB to mid-log phase, cells were harvested and re-suspended in PBS containing 1% starch (Difco) and 1% glucose to extract the βh/c activity from the bacterial surface. After incubation for 1 hour at 37°C, the supernatant was filtered and added 1∶1 (vol/vol) to 100% ice cold methanol, and incubated for a further 60 min at 4°C. The supernatant was then centrifuged and the pellet was re-suspended in PBS. 10/84 and 10/84Δ*cylE* extracts (designated hereafter βh/c^e^ and ΔcylE^e^) were filtered and assayed for hemolytic activity by sheep red blood cell (SRBC) lysis assay and colorimetric quantification of hemoglobin release [25].

### LLO purification

Plasmid vector encoding recombinant His-tagged LLO was a generous gift from Dr. D. Portnoy (University of California, Berkeley) and was purified as follows; E. *coli* strain BL21 (λDE3) pLysS (Promega) were transformed with a C-terminally 6-histidine tagged version of the LLO cDNA in the pET29b vector. Bacteria from a single colony were grown in LB broth for 16 h at 30°C, then diluted and expanded at 30°C to log phase, at which point expression of LLO was induced by the addition of isopropyl-1-thio-β-D-galactopyranoside (IPTG, Invitrogen) to a final concentration of 1 mM for 4 h. Cells were collected by centrifugation and lysed using a French press (Thermo Spectronic, Rochester, NY). The lysate was centrifuged at 12,000× g for 40 min and the supernatant incubated with Ni-NTA agarose (Qiagen) for 1 h. The agarose was extensively washed and the His-tagged LLO eluted with 400 mM imidazole (Sigma-Aldrich). The eluent was dialyzed against TBS (10 mM Tris-Cl, 140 mM NaCl, pH 8.5 at 4°C), and the activity of purified LLO was confirmed by SRBC lysis assay. The molecular weight and relative purity of recombinant LLO was confirmed by SDS-PAGE and coomassie staining, protein concentration was determined by Bradford assay (Biorad).

### Macrophage killing assay

Log-phase GBS were added to BMDM and brought into contact with cells by centrifugation at 600 g for 5 min. Gentamycin (50 µg/ml) was added to the culture after 10 min, and cells incubated for a 60–120 min at 37°C in 5% CO_2_. Cells were washed and lysed in 0.02% Triton X-100 in PBS. Serial dilutions of the lysates were plated on THA in triplicate and incubated at 37°C for enumeration of cfu.

### Cell viability assay

Cell viability was monitored by MTT assay. MTT (Merck) was dissolved in PBS (5 mg/ml), filtered and stored at −20°C until use. MTT solution was added to the cell culture medium at 10% of the culture volume and the supernatants were gently removed after 30 min. The formazan crystals were dissolved in DMSO and their absorbance was measured at 540 nm using a spectrophotometer.

### Gene expression analysis

Total cellular RNA was extracted using QIAamp RNA Blood Mini Kit (Qiagen) and stored at −80°C before analysis by real-time quantitative PCR (qPCR) or ribonuclease protection assay (RPA). For qPCR analysis; cDNA was synthesised from 0.5 µg of DNase treated total RNA using Moloney murine leukaemia virus (M-MLV) reverse transcriptase following the manufacturer's instructions (Promega). mRNA expression was quantified using sequence specific primers in the presence of SYBR Green PCR Master Mix using an ABI Prism 7700 thermocycler (Applied Biosystems). Primer sequences are available upon request. Reactions were performed in duplicate or triplicate and mRNA expression normalized to levels of cyclophillin mRNA. For RNase protection assays; 0.5 µg of total RNA was hybridized with ^32^P-α-UTP-labeled multi-probe RNA templates (BD Biosciences, Riboquant). After RNAse treatment and purification of protected probes, samples were separated by electropheresis on 6% denaturing SDS gel and analysed by autoradiography.

### Kinase assay and immunoblotting

Whole-cell protein extracts were prepared using lysis buffer containing 50 mM HEPES pH 7.9, 250 mM NaCl_2_, 3 mM EDTA pH8, 3 mM EGTA, 1% Triton X-100, 10% glycerol, 1∶100 protease/phosphatase inhibitor cocktail (Sigma Aldrich). Specific kinase activities were measured after immunoprecipitation (IP) with either; anti-IKKγ antibody (ab764, BD Biosciences), anti-JNK or anti-p38 antibody (both gifted by Prof J Saklatvala, Imperial College, London, UK), immune-complexes were incubated in the presence of ^32^P-γ-ATP for 30 min at RT in a kinase buffer containing 20 mM HEPES pH 7.5, 10 mM MgCl_2_, 1 mM dithiothreitol (DTT) together with the following recombinant substrates; GST-cJun^1–79^, GST-MAPKAPK2 (GST-MK2^1–150^) or GST-IκBα^1–54^. JNK, p38 and IKK recovery was determined by immunoblotting (IB) with anti-IKKα antibody (M280, Santa Cruz Biotechnology), anti-JNK1 antibody (BD Biosciences) or anti-p38 antibody (Cell Signaling). Reactions were denatured then resolved by SDS-PAGE and transferred to polyvinylidene difluoride (PVDF) membranes (Perkin Elmer Life Science inc) by electroblotting. Substrate phosphorylation was revealed by autoradiography followed by phosphor imager quantification (Fuji FLA-2000). Immunoblotting was performed on SDS-PAGE fractionated protein extracts according to standard protocols.

### Cytokine-ELISA

Cell-free supernatants were analyzed for IL-10, IL12p40 and TNF-α content by sandwich ELISA (BD Biosciences, OptEIA) according to the manufacturer's instructions.

### Statistical analysis

Quantitative *in vitro* cell based assays were analysed using ANOVA and p values indicated. *In vivo* experiments were analysed using non-parametric Mann Whitney t-test and p values are indicated where appropriate.

### Ethics statement

Animal experimentation was conducted in strict accordance with good animal practice as defined by the French animal welfare bodies relative to European Convention (EEC Directive 86/609) and approved by the Direction Départmentale des Services Vétérinaires des Bouches du Rhônes.

## Supporting Information

Figure S1 **A**. βh/c^e^ and ΔcylE^e^ extracts were added to macrophages at a dilution of 1∶200 and protein extracts were prepared at the indicated time points. JNK, p38 and IKK activation was measured IP kinase assay, as described in Fig. 2. **B**. Macrophages were infected with wt GBS (10/84, MOI: 5) alone, or 10/84Δ*cylE* bacteria (MOI: 5) in the presence of βh/c^e^ or ΔcylE^e^ extracts (1∶200). Protein extracts were prepared at the indicated time points and JNK, p38 and IKK activity measured by IP kinase assay, as described in Fig. 2. **C**. Viability of macrophages in the presence of βh/c^e^ and ΔcylE^e^ extracts at the indicated dilutions was quantified by MTT assay after 8 h. Data is represented as mean ± sem of 4 replicates. **D**. Macrophage viability after stimulation with heat-killed 10/84Δ*cylE* bacteria (h10/84Δ) in the presence of βh/c^e^ or ΔcylE^e^ (1∶200). Data is represented as mean ± sem of 4 replicates. Representative data of at least 2 independent experiments is shown.
(TIFF)

Figure S2 Primary macrophages were prepared from wild type (Wt) and knockout mice for; TLR4 (Tlr4−/−; A), NOD2 (Nod2−/−; B), Caspase-1 (Casp1−/−; C) and ASC (Asc1−/−; D). Macrophages were infected with 10/84 (MOI: 5) and protein extracts prepared at the indicated time points. JNK, p38 and IKK activity were measured by IP kinase assay, as described in Fig. 2. IB analysis of IKKα was used as loading control.
(TIFF)

Figure S3 **A**. Macrophages derived from Wt and IL-10 knockout mice (Il10−/−) were infected with 10/84 (MOI: 5) and total RNA isolated at the indicated time points, IL-12p40 mRNA was measured by qPCR and data expressed as fold induction normalized to cyclophillin mRNA. **B**. Wt macrophages were stimulated with h10/84Δ and βh/c^e^ (1∶200) in the presence or absence of 1 µg/ml anti-mouse IL-10 receptor-blocking antibody (αIL-10r ab), total RNA was prepared at the indicated time points and IL-12p40 expression measured by qPCR, as described above. **C**. Total RNA was isolated from p38^f/f^ and p38^ΔMye^ macrophages infected with 10/84 (MOI: 5) at the indicated time points and analyzed by RPA using a multi-probe template set to measure cytokine mRNA expression. Representative data of at least 2 independent experiments is shown.
(TIFF)

Figure S4 Macrophages were collected from the peritoneal cavity of p38^f/f^ and p38^ΔMye^ mice infected i.p. with 5×10^7^ cfu GBS (10/84) after 8 h and isolated by adherence to plastic for 2 h *in vitro*. Total RNA was prepared from adherent cells and IL-10 mRNA measured by qPCR, data is expressed as relative mRNA expression normalized to cyclophillin mRNA. Statistical analysis was performed using non-parametric Mann Whitney t-test, p = 0.072.
(TIFF)

Figure S5 **A**. Macrophages were treated with 2.5 µg/ml saponin with and without stimulation with heat-killed 10/84Δ*cylE* bacteria (h10/84Δ) and protein extracts were prepared at the indicated time points. IKK and p38 activity was measured by IP kinase assay, as described in Fig. 2. IB analysis of IKKα was used a loading control. **B**. Total RNA was isolated from macrophages stimulated with h10/84Δ in the presence and absence of saponin (2.5 µg/ml) at the indicated time points. IL-10 and IL-12p40 mRNA expression was measured by qPCR and normalized to cyclophillin mRNA. Data is expressed as fold induction and mean ± sem of 3 replicates is plotted. Representative data of at least 2 independent experiments is shown.
(TIFF)
