# Supplementary figures and images for: The Pore-Forming Toxin β hemolysin/cytolysin Triggers p38 MAPK-Dependent IL-10 Production in Macrophages and Inhibits Innate Immunity
Source: PLoS Pathog. 2012 Jul 19;8(7):e1002812. doi: 10.1371/journal.ppat.1002812 (PMC3400567; doi:10.1371/journal.ppat.1002812)

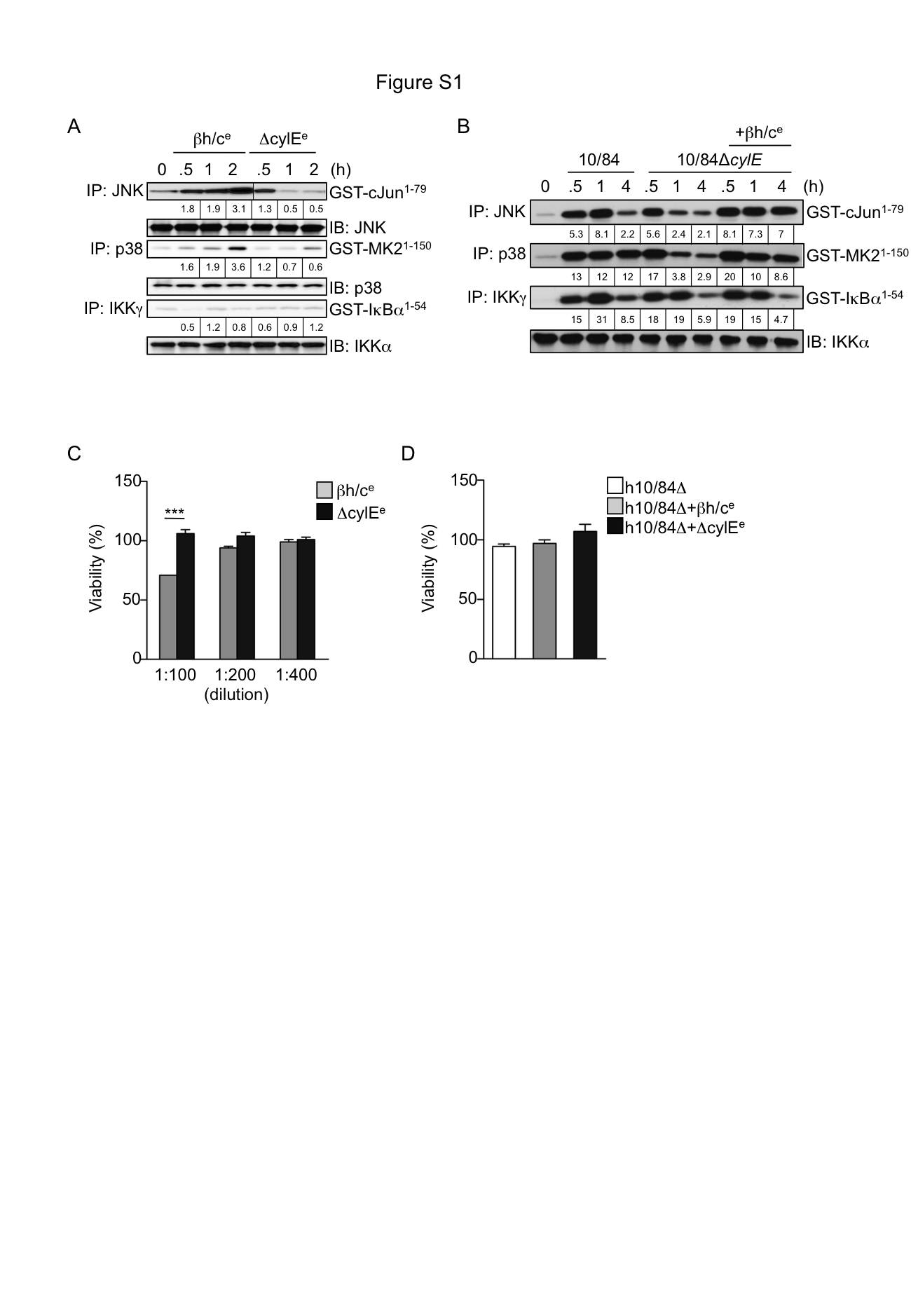

Supplement: Figure S1 — A. βh/ce and ΔcylEe extracts were added to macrophages at a dilution of 1∶200 and protein extracts were prepared at the indicated time points. JNK, p38 and IKK activation was measured IP kinase assay, as described in Fig. 2. B. Macrophages were infected with wt GBS (10/84, MOI: 5) alone, or 10/84ΔcylE bacteria (MOI: 5) in the presence of βh/ce or ΔcylEe extracts (1∶200). Protein extracts were prepared at the indicated time points and JNK, p38 and IKK activity measured by IP kinase assay, as described in Fig. 2. C. Viability of macrophages in the presence of βh/ce and ΔcylEe extracts at the indicated dilutions was quantified by MTT assay after 8 h. Data is represented as mean ± sem of 4 replicates. D. Macrophage viability after stimulation with heat-killed 10/84ΔcylE bacteria (h10/84Δ) in the presence of βh/ce or ΔcylEe (1∶200). Data is represented as mean ± sem of 4 replicates. Representative data of at least 2 independent experiments is shown. (TIFF) [file ppat.1002812.s001.tiff]

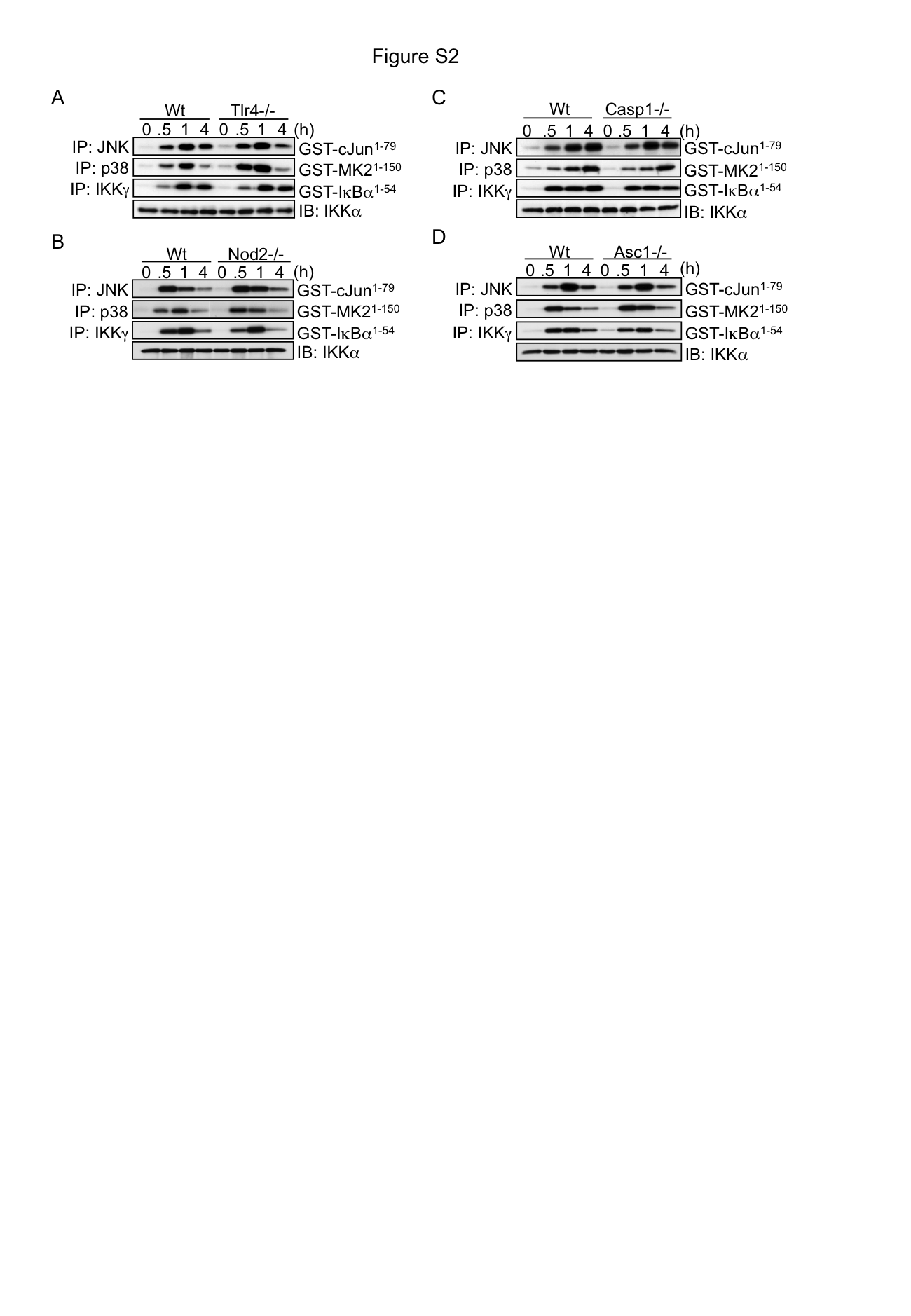

Supplement: Figure S2 — Primary macrophages were prepared from wild type (Wt) and knockout mice for; TLR4 (Tlr4−/−; A), NOD2 (Nod2−/−; B), Caspase-1 (Casp1−/−; C) and ASC (Asc1−/−; D). Macrophages were infected with 10/84 (MOI: 5) and protein extracts prepared at the indicated time points. JNK, p38 and IKK activity were measured by IP kinase assay, as described in Fig. 2. IB analysis of IKKα was used as loading control. (TIFF) [file ppat.1002812.s002.tiff]

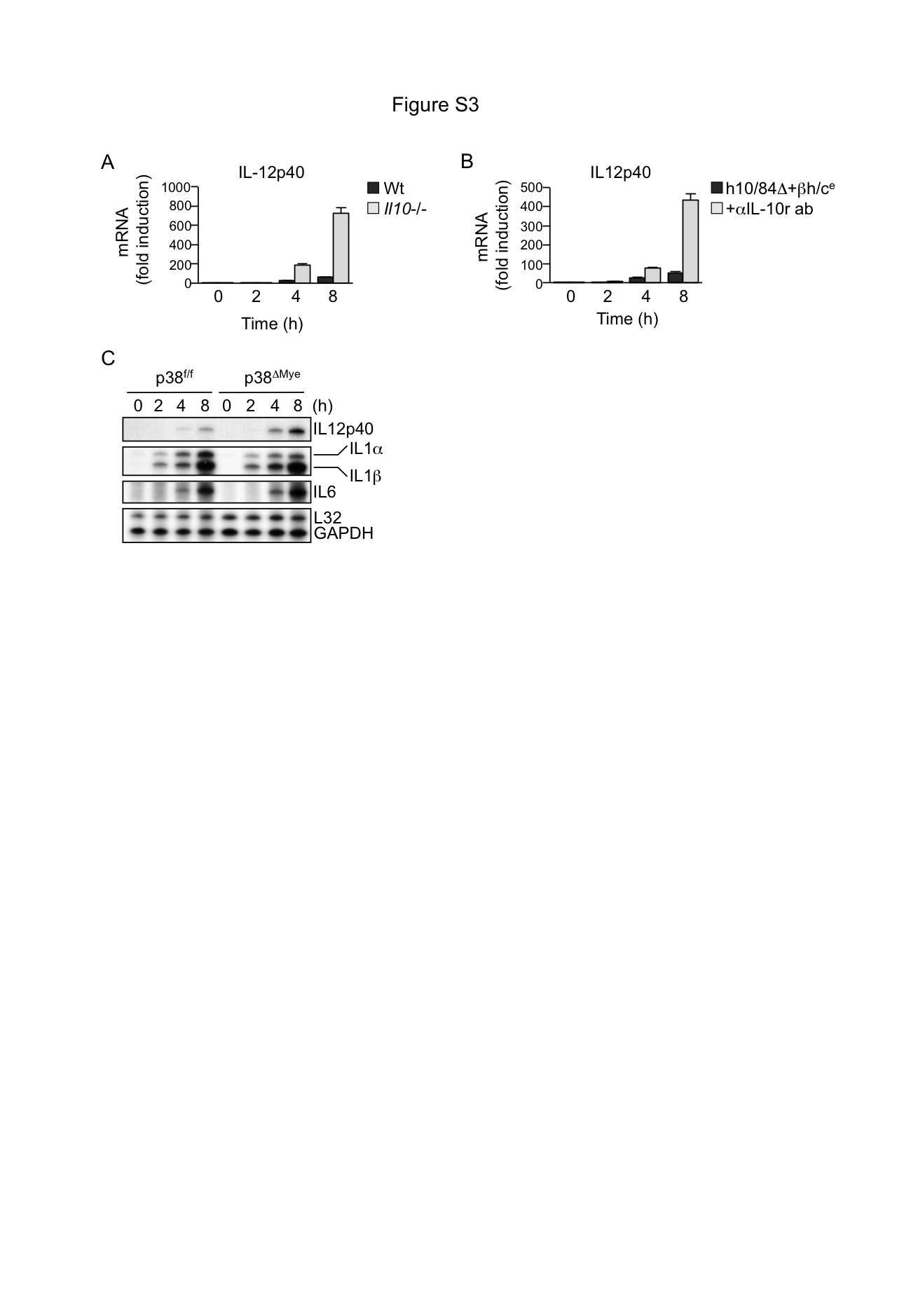

Supplement: Figure S3 — A. Macrophages derived from Wt and IL-10 knockout mice (Il10−/−) were infected with 10/84 (MOI: 5) and total RNA isolated at the indicated time points, IL-12p40 mRNA was measured by qPCR and data expressed as fold induction normalized to cyclophillin mRNA. B. Wt macrophages were stimulated with h10/84Δ and βh/ce (1∶200) in the presence or absence of 1 µg/ml anti-mouse IL-10 receptor-blocking antibody (αIL-10r ab), total RNA was prepared at the indicated time points and IL-12p40 expression measured by qPCR, as described above. C. Total RNA was isolated from p38f/f and p38ΔMye macrophages infected with 10/84 (MOI: 5) at the indicated time points and analyzed by RPA using a multi-probe template set to measure cytokine mRNA expression. Representative data of at least 2 independent experiments is shown. (TIFF) [file ppat.1002812.s003.tiff]

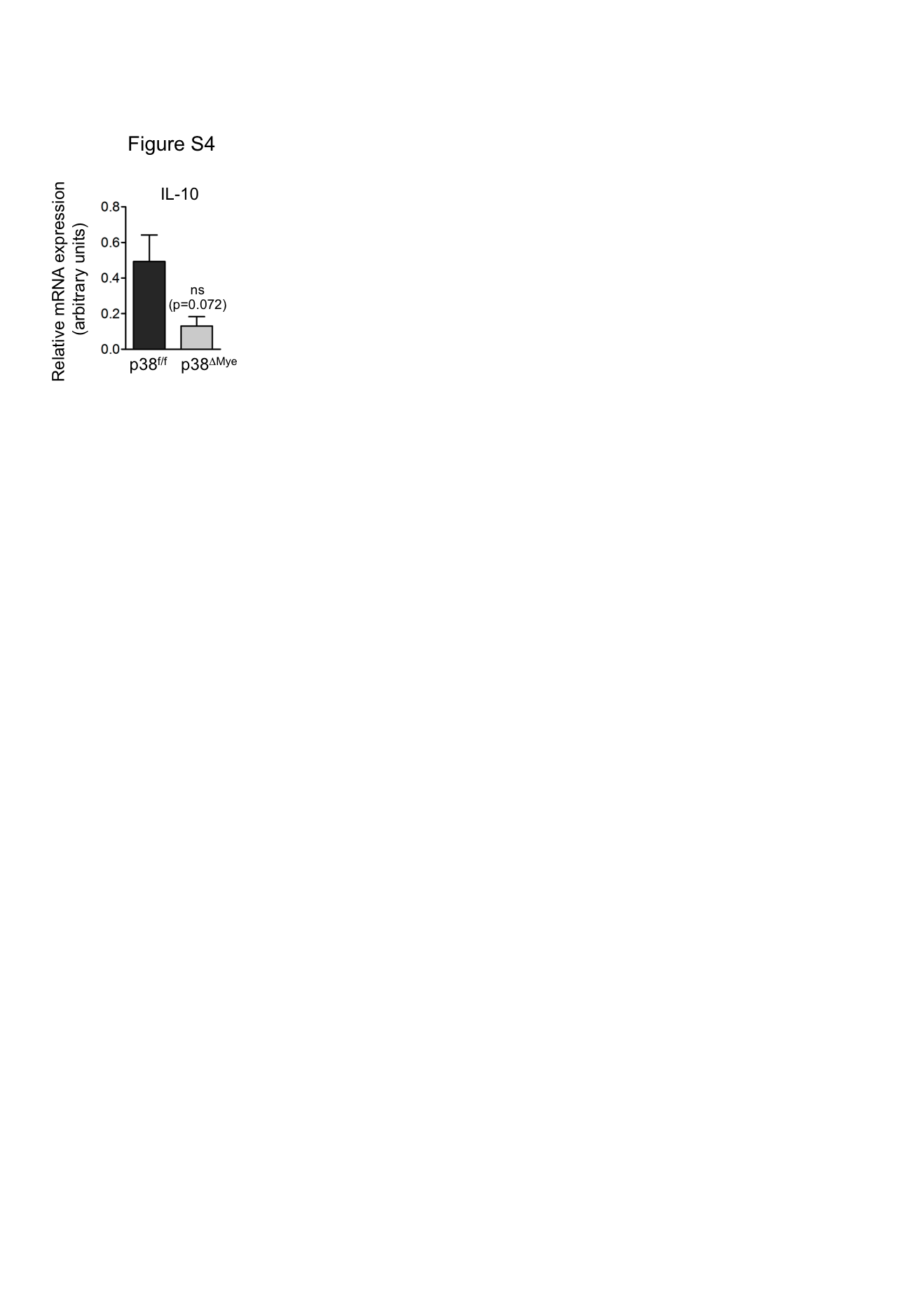

Supplement: Figure S4 — Macrophages were collected from the peritoneal cavity of p38f/f and p38ΔMye mice infected i.p. with 5×10^7^ cfu GBS (10/84) after 8 h and isolated by adherence to plastic for 2 h in vitro. Total RNA was prepared from adherent cells and IL-10 mRNA measured by qPCR, data is expressed as relative mRNA expression normalized to cyclophillin mRNA. Statistical analysis was performed using non-parametric Mann Whitney t-test, p = 0.072. (TIFF) [file ppat.1002812.s004.tiff]

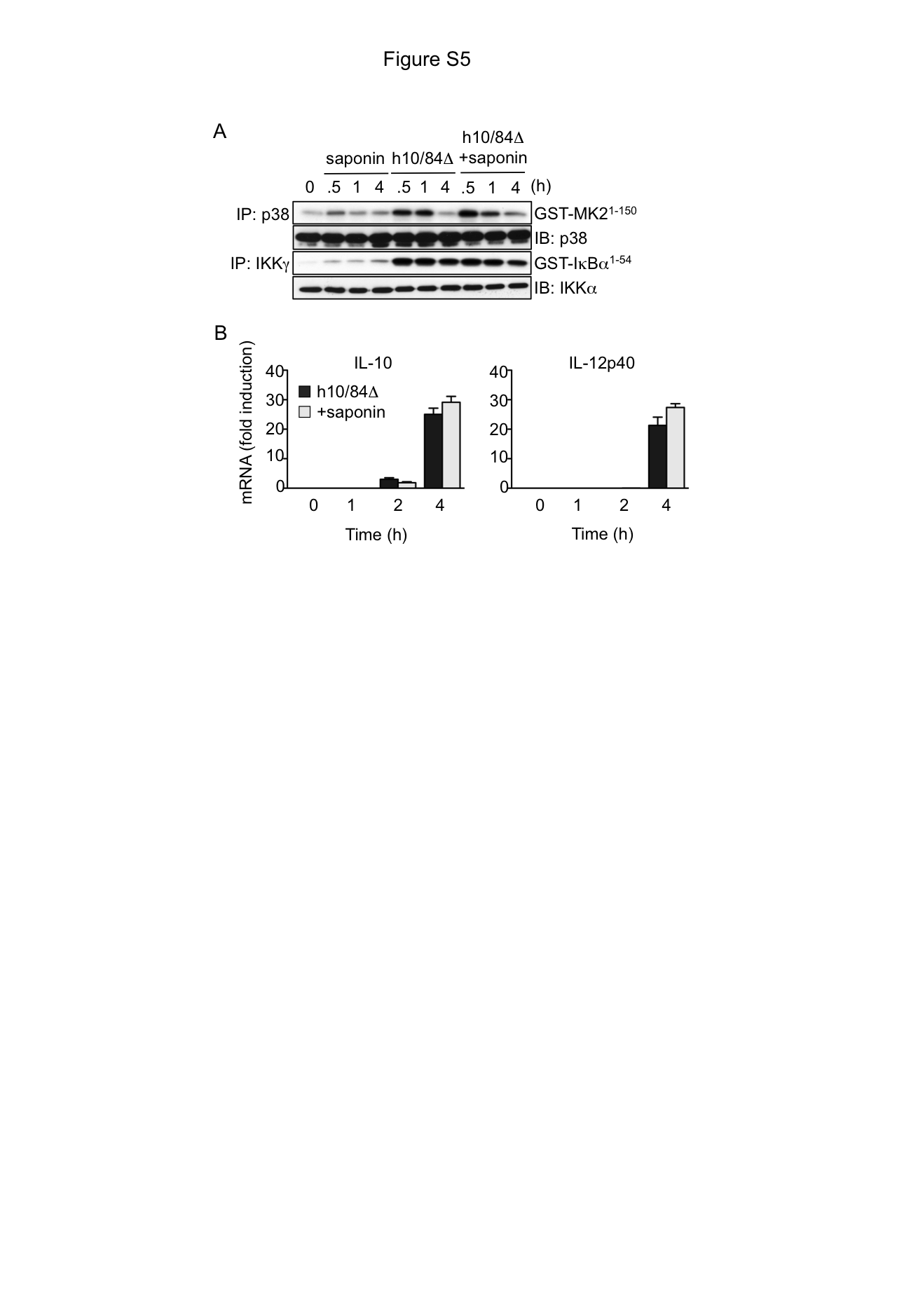

Supplement: Figure S5 — A. Macrophages were treated with 2.5 µg/ml saponin with and without stimulation with heat-killed 10/84ΔcylE bacteria (h10/84Δ) and protein extracts were prepared at the indicated time points. IKK and p38 activity was measured by IP kinase assay, as described in Fig. 2. IB analysis of IKKα was used a loading control. B. Total RNA was isolated from macrophages stimulated with h10/84Δ in the presence and absence of saponin (2.5 µg/ml) at the indicated time points. IL-10 and IL-12p40 mRNA expression was measured by qPCR and normalized to cyclophillin mRNA. Data is expressed as fold induction and mean ± sem of 3 replicates is plotted. Representative data of at least 2 independent experiments is shown. (TIFF) [file ppat.1002812.s005.tiff]
